# Silver Nanoparticles at Low Concentrations Embedded in ECM Promote Endothelial Monolayer Formation and Cell Migration

**DOI:** 10.3390/ijms26104761

**Published:** 2025-05-16

**Authors:** Barbara Wójcik, Katarzyna Zawadzka, Anna Hotowy, Maria Jóźwiak, Klaudia Jusińska, Mateusz Wierzbicki

**Affiliations:** Department of Nanobiotechnology, Warsaw University of Life Science, Ciszewskiego 8, 02-786 Warsaw, Poland; katarzyna_zawadzka1@sggw.edu.pl (K.Z.); anna_hotowy@sggw.edu.pl (A.H.); maria.jozwiak199@gmail.com (M.J.); s200008@sggw.edu.pl (K.J.); mateusz_wierzbicki@sggw.edu.pl (M.W.)

**Keywords:** HUVEC, ECM, AgNPs, migration, cytotoxicity

## Abstract

Several scientific studies have reported the opposing effects of silver nanoparticles (AgNPs) on angiogenesis, ranging from proangiogenic to anti-angiogenic. The widespread use of AgNPs in biomedical applications and the variability of their effects depending on concentration and exposure conditions highlight the need for further research into their impact on vascularization and endothelial cell behavior. This study aimed to investigate the potential influence of AgNPs on human umbilical vein endothelial cells (HUVECs) using a model incorporating a thin layer of an extracellular matrix (ECM). To this end, cytotoxicity was assessed, and endogenous nitric oxide and superoxide levels were measured. Additionally, the effects of AgNPs on HUVEC confluence and migration were evaluated. The expression levels of 43 proteins involved in angiogenesis were also analyzed. The results revealed that ECM enriched with AgNPs at a concentration of 0.5 mg/L enhanced cell coverage, promoted migration, and supported monolayer formation without inducing cytotoxicity.

## 1. Introduction

Blood vessels are essential for maintaining homeostasis, transporting oxygen, nutrients, signaling molecules, and metabolic byproducts [1]. Angiogenesis—the formation of new blood vessels—occurs in both physiological and pathological processes in the human body. It plays a crucial role in wound healing and immune system homeostasis and is tightly regulated through complex interactions with serum components and the extracellular matrix (ECM) [2].

According to the literature, endothelial cell migration is stimulated not only by specific cytokines, such as vascular endothelial growth factor (VEGF), fibroblast growth factor (FGF), angiopoietins, and transforming growth factor-beta (TGF-β), but also by the organization of the ECM within the surrounding microenvironment [2]. The ECM is critical in regulating angiogenesis, as it provides structural support, acts as a scaffold for cytokines, and participates in direct signaling functions [3]. Notably, endothelial cell adhesion to the ECM via integrins is essential for the activation of Erk1/Erk2/MAP kinase signaling pathways [4], which promote cell proliferation and angiogenesis [5] and inhibit apoptosis [6]. Furthermore, endothelial cells’ migration also depends on their interaction with the ECM [7]. ECM components, such as fibrin and collagen I, have been shown to support cytokine-induced migration. Additionally, certain molecules immobilized within the ECM can induce haptotactic migration independently of cytokine stimulation [8].

The unique properties of silver nanoparticles (AgNPs) make them promising candidates for applications in the biomedical, food, and environmental industries [9]. AgNPs induce various oxidative and non-oxidative processes, including surface adhesion, electrostatic interactions with cellular membranes, cell destruction, organelle dysfunction, and interactions with proteins and nucleic acids. They are widely used as antifungal, antibacterial, and antiviral agents [10,11], in wound dressings, long-term burn care products [12], medical device coatings, medical textiles, orthopedic materials, contraceptive devices, cosmetic fabrics, lotions for therapeutic and supplementary drug or nutrient delivery, paints, sunscreens, and more [13,14,15]. However, the extensive use of Ag nanostructures also presents several challenges, such as instability, binding to major blood proteins, the potential to damage proteins, nucleic acids, and membranes, as well as the immunosuppression of various cytokines [16]. AgNPs exhibit significant dose-dependent effects, particularly in terms of toxicity. Both in vitro and in vivo evaluations have demonstrated that fibronectin-coated AgNPs may serve as novel antimicrobial and anti-inflammatory surface modifications for vascular repair and regenerative engineering. Given the limited availability of endothelial cells, achieving optimal biological performance, the superior biocompatibility of synthetic grafts, and a high endothelialization capacity is critical for reducing immune responses and ensuring long-term implant success [17]. Therefore, it is essential to determine the appropriate concentration range of AgNPs in the extracellular matrix that could be safely and effectively used in wound dressings or biological scaffolds for tissue engineering to stimulate endothelial cells and promote blood vessel formation.

This study aimed to investigate the potential influence of AgNPs on HUVECs using a model containing a thin layer of ECM. We hypothesize that AgNPs embedded in the ECM modulate endothelial cell behavior, including proliferation and migration, and influence angiogenic signaling by altering the expression of angiogenesis-related proteins, as well as the levels of nitric oxide (NO) and superoxide.

## 2. Results

### 2.1. Physicochemical Analysis of AgNPs

**Figure 1:**
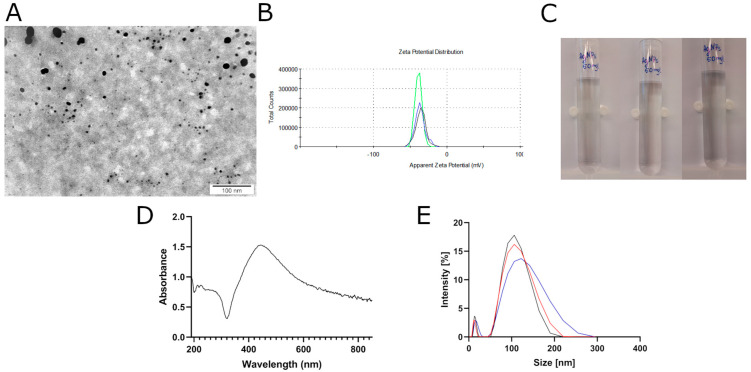
Physiochemical analysis of silver nanoparticles (AgNPs). (**A**) Morphology of AgNPs visualized by transmission electron microscopy (TEM). (**B**) Zeta potential distribution of AgNPs. (**C**) Photographs of the hydrocolloid after 0 h, 24 h, and 48 h. (**D**) UV–Vis spectrum of AgNPs. (**E**) Hydrodynamic diameter of AgNPs−size distribution.

Transmission electron microscopy (TEM) was used to visualize the morphology of AgNPs. The analysis revealed that the metallic nanoparticles were spherical (Figure 1A) and exhibited size heterogeneity. The diameters of the AgNPs ranged from 4 to 26 nm. The zeta potential of the AgNPs was negative, with a value of −36.63 ± 1.37 mV (Figure 1B). The hydrodynamic diameter of AgNPs in hydrocolloid was at the level of 114.8 ± 8.56 nm (Figure 1E). Furthermore, an analysis of the UV–Vis spectrum revealed the highest peak at the wavelength of 443.5 nm (Figure 1D). After 48 h, the hydrocolloid of AgNPs remained clear without visible sediment (Figure 1C).

### 2.2. Analysis of Nanoparticles Toxicity

**Figure 2:**
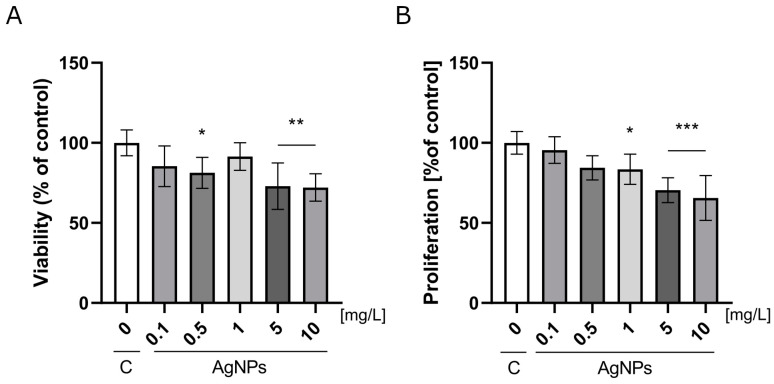
Analysis of silver nanoparticle (AgNP) toxicity. (**A**) The viability of HUVECs after a 24 h incubation on nanoplatforms composed of a Geltrex ECM and AgNPs at concentrations ranging from 0.1 to 10 mg/L. (**B**) The proliferation of HUVECs after 48 h of incubation on the same nanoplatforms. The results are presented as a percentage of the control group ± standard deviation. Asterisks above the bars indicate statistically significant differences compared to the control (* *p* < 0.05; ** *p* ≤ 0.01; *** *p* < 0.001).

Cell viability and proliferation assays were performed to assess the cytotoxicity of nanoplatforms composed of AgNPs embedded in a Geltrex ECM. The results are presented in Figure 2. The viability assay revealed a reduction in HUVEC viability after 24 h of incubation on nanoplatforms containing AgNPs at a concentration of 0.5 mg/L, with cell viability reaching 81.25% relative to the control. Further increases in AgNP concentration to 5 and 10 mg/L resulted in a more pronounced decrease in viability, dropping to 73% and 72% of the control, respectively (Figure 2A). Similarly, the incorporation of AgNPs into the ECM at concentrations ranging from 1 to 10 mg/L led to a dose-dependent inhibition of cell proliferation after 48 h. HUVEC proliferation declined progressively, ranging from 83% to 66% of the control at the highest concentration tested (Figure 2B).

### 2.3. Nitric Oxide and Mitochondrial Superoxide Production

**Figure 3:**
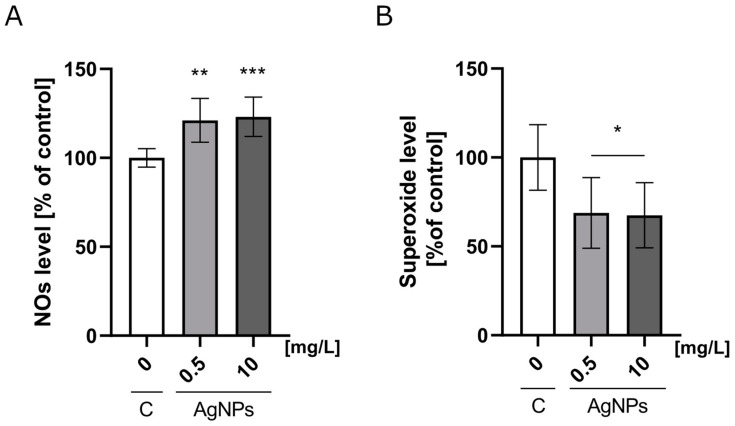
Nitric oxide and mitochondrial superoxide synthesis levels. (**A**) Nitric oxide and (**B**) mitochondrial superoxide levels in HUVECs after 24 h of incubation on nanoplatforms composed of Geltrex ECM and AgNPs at concentrations of 0.5 mg/L and 10 mg/L. The results are presented as a percentage of the control ± standard deviation. Asterisks above the bars indicate statistically significant differences compared to the control (* *p* < 0.05; ** *p* ≤ 0.01; *** *p* < 0.001).

A Nitric Oxide Synthase Detection System was used to analyze the nitric oxide (NO) level. The analysis of NO levels in HUVECs cultured on the nanoplatforms revealed that the addition of AgNPs stimulated NO production in a dose-dependent manner. The NO level increased to 121% of the control on nanoplatforms containing 0.5 mg/L AgNPs and rose to 123% when the concentration increased to 10 mg/L (Figure 3A). Interestingly, the incorporation of AgNPs into the Geltrex ECM nanoplatforms resulted in a decrease in mitochondrial superoxide levels. At the highest tested concentration of AgNPs (10 mg/L), the superoxide level dropped to 67% of the control (Figure 3B).

### 2.4. HUVEC Confluence Level Analysis

**Figure 4:**
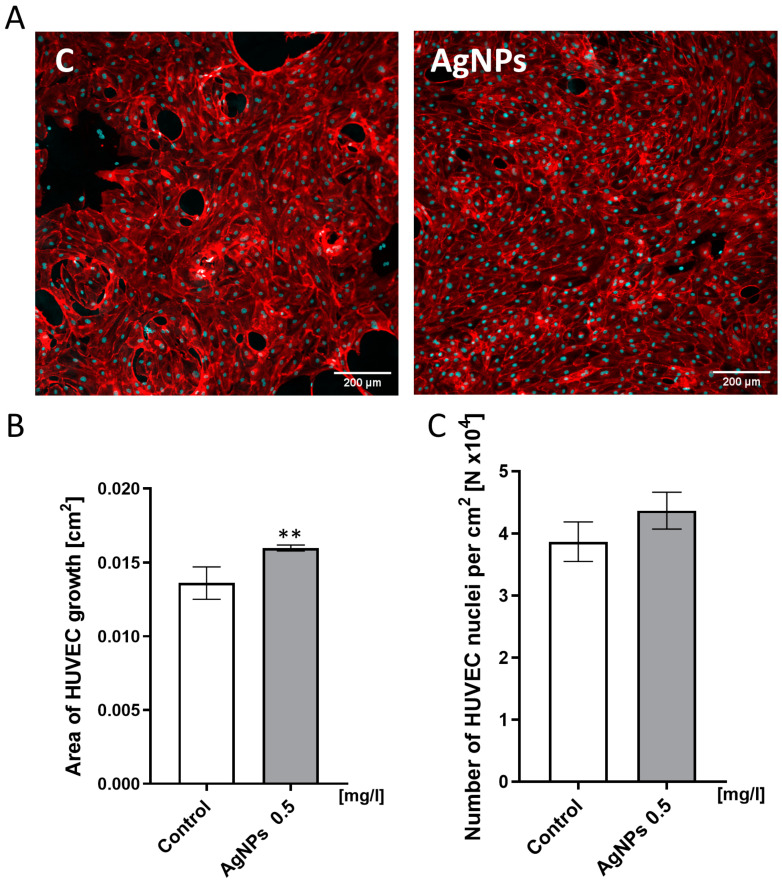
HUVEC monolayer formation. (**A**) Confocal images of HUVECs cultured on control Geltrex ECM (“C”) and ECM supplemented with AgNPs at 0.5 mg/L (“AgNPs”). The Geltrex ECM enriched with a low concentration of AgNPs increased the cell coverage and promoted monolayer formation. (**B**) The quantitative analysis of the cell-covered growth area. (**C**) The quantification of the number of cell nuclei per cm^2^. The nuclei were stained with Hoechst 33,342 (cyan color), and the actin cytoskeleton was labeled with Atto 633-conjugated phalloidin (red color). Measurements were performed using ImageJ software (version 1.54p). The results are presented as mean ± standard deviation. Asterisks above the bars indicate statistically significant differences between control and experimental groups (** *p* ≤ 0.01).

To evaluate the effect of an AgNP-enriched ECM on endothelial cell coverage and monolayer formation, HUVEC confluence was analyzed using confocal microscopy and image-based quantification. The analysis of HUVEC confluence revealed that the Geltrex ECM enriched with AgNPs at a concentration of 0.5 mg/L promoted more stable monolayer formation (Figure 4A). Interestingly, the same number of cells (Figure 4C) covered a larger growth area when cultured on the AgNP-modified ECM (Figure 4B). Specifically, the cells on an unmodified ECM covered an average area of 0.014 cm^2^, whereas the cells on the ECM supplemented with 0.5 mg/L AgNPs covered 0.016 cm^2^.

The analysis of HUVEC confluence revealed that the Geltrex ECM enriched with AgNPs at the concentration of 0.5 mg/L promoted more stable monolayer formation (Figure 4A). Moreover, the same number of cells (Figure 4C) covered a larger growth area (Figure 4B). As shown in Figure 4B, the cells harvested from the ECM covered 0.014 cm^2^ of growth area. In contrast, the cells harvested from the Geltrex ECM with AgNPs added at a concentration of 0.5 mg/L covered 0.016 cm^2^ of growth area.

### 2.5. Migration Analysis

**Figure 5:**
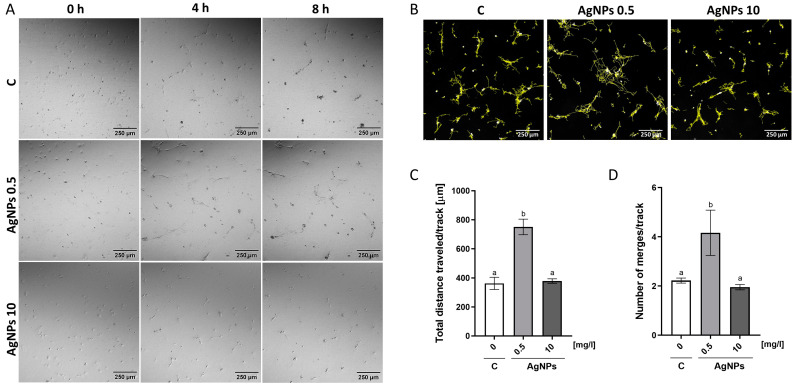
Analysis of HUVEC migration on ECM. (**A**) Morphological characterization of HUVECs on control ECM (“C”) and ECM supplemented with AgNPs at 0.5 mg/L and 10 mg/L, shown at 0 h, 4 h, and 8 h. (**B**) Migration tracks of the cells cultured on the control ECM and the ECM with 0.5 mg/L and 10 mg/L AgNPs, visualized using the TrackMate plugin in ImageJ. (**C**) Graph showing the quantification data of the total distance traveled per cell track. (**D**) The quantification of the number of merges per track. All the data are presented as the mean ± standard deviation. Different letters above the bars indicate statistically significant differences between groups (*p* < 0.05).

Time-lapse imaging and cell tracking were used to analyze HUVEC migration behavior to assess the impact of an AgNP-enriched ECM on endothelial cell motility. The analysis revealed that the addition of AgNPs at a concentration of 0.5 mg/L to the Geltrex ECM significantly enhanced HUVEC migration (Figure 5). The mean distance traveled by cells in this group was 751 µm, compared to 362 µm in the control group. Additionally, the number of merges per track in the 0.5 mg/L AgNP group was approximately twice as high as in the control. In contrast, the ECM containing AgNPs at a concentration of 10 mg/L did not result in statistically significant differences in the total distance traveled or the number of merges per track.

### 2.6. Antibody Array Analysis

**Figure 6:**
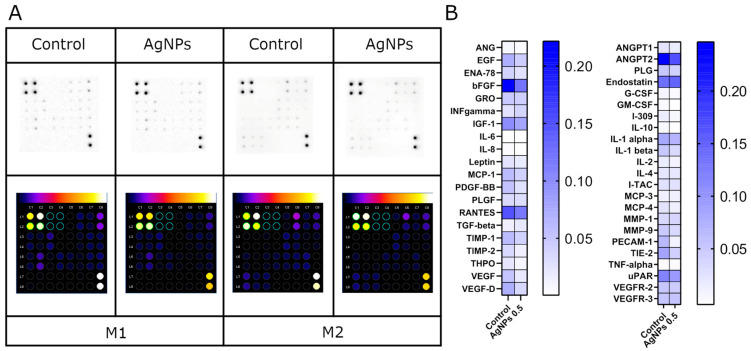
Proteins involved in angiogenesis expressed after 24 h incubation of HUVECs on nanoplatforms made of Geltrex ECM (control) and ECM with AgNPs at concentration of 0.5 mg/L, determined by human angiogenesis antibody array membranes. The results were normalized to the control groups for M1 and M2. Images were created with ImageJ software (**A**). Mean chemiluminescence value for each protein (**B**).

The next analysis to be performed was the human angiogenesis antibody array. The level of 43 proteins involved in angiogenesis was measured. In this analysis, only the influence of the addition of Ag at a concentration of 0.5 mg/L was tested. The location of the proteins on the membranes was presented in Tables S1 and S2, which are included in the Supplementary Data. The analysis revealed that enrichment of the Geltrex ECM with AgNPs resulted in decreased levels of the following proteins: basic fibroblast growth factor (bFGF); regulated on activation, normal T-cell expressed and secreted chemokine (RANTES); vascular endothelial growth factor D (VEGF-D); platelet and endothelial cell adhesion molecule 1 (PECAM-1); angiopoetin-1 receptor (TIE-2); and urokinase plasminogen activator surface receptor (uPAR). Moreover, adding AgNPs into the ECM nanoplatform caused an elevated endostatin level (Figure 6).

## 3. Discussion

The potential use of silver nanoparticles (AgNPs) has gained widespread interest, with numerous studies published on AgNP-induced cytotoxicity. However, few have explored the effects of low-dose AgNPs, and even fewer have considered the influence of the surrounding extracellular matrix (ECM) on their cytotoxicity. Given the significant role of the ECM in angiogenesis-related processes, our analyses employed a model incorporating the Geltrex LDEV-Free Reduced Growth Factor Basement Membrane Matrix.

Our findings indicate that low concentrations of metallic nanoparticles can induce cytotoxicity in HUVECs. Interestingly, 24 h after seeding the cells on nanoplatforms composed of the ECM and AgNPs at a concentration of 0.5 mg/L, a reduction in viability was observed. However, this did not affect proliferation, which remained comparable to the control group. Conversely, the ECM’s supplementation with AgNPs at 1 mg/L caused the opposite effect, decreasing proliferation as the AgNP concentration increased. This suggests that even low doses of AgNPs may compromise endothelial barrier integrity. Increased permeability facilitates macromolecular movement [18].

The cellular localization of AgNPs is strongly influenced by their size, aggregation state, and surface characteristics. To assess this, hydrodynamic diameter measurements were performed, revealing an average agglomerate size of 114.8 nm, while individual nanoparticles ranged from 4 to 26 nm in diameter. According to the existing literature, AgNPs are predominantly internalized via endocytosis [19], although non-aggregated nanoparticles may also enter cells through direct membrane translocation mechanisms, such as the flip-flop mechanism or via ion channels [20]. Uptake efficiency is size-dependent, with nanoparticles of 5, 20, 50, and 100 nm showing internalization rates of 40.3%, 22.0%, 52.3%, and 76.2%, respectively [21]. Once internalized, AgNPs are generally localized within membrane-bound compartments. Particles of between 20 and 100 nm typically accumulate in early endosomes that subsequently mature into late endosomes [21,22]. In some cases, AgNPs are detected in the cytosol, which may result from endosomal escape caused by membrane disruption or ionic imbalances [23]. Nuclear entry is largely determined by particle size relative to the nuclear pore complex, which allows passive diffusion of particles up to approximately 50 nm, depending on the cell type [24]. Several studies have reported the nuclear localization of AgNPs smaller than 50 nm [25,26].

Upon endocytosis, AgNPs are primarily translocated to lysosomes and endosomes [27]. Depending on their size, internalized nanoparticles may also be located in mitochondria and the nucleus [28]. Inside the cell, AgNPs can trigger a cascade of events, including cell membrane permeabilization, reactive oxygen species (ROS) production, inflammatory responses, DNA damage, chromosomal aberrations, and apoptosis [29,30]. Several mechanisms underlie the cytotoxicity of silver nanoparticles (AgNPs). One involves mitochondrial dysfunction resulting from the intracellular accumulation of Ag^+^ ions and Ag^0^ species produced via oxidative processes [31]. Another proposed mechanism is the interaction of AgNPs with membrane proteins, leading to the generation of reactive oxygen species (ROS). Due to silver’s strong affinity for sulfur-containing groups, this interaction can result in damage to nucleic acids and proteins, ultimately inhibiting proliferation and triggering apoptosis [32].

Our results are consistent with findings in CHO-K1 cell lines, where AgNPs at 5 and 10 mg/L reduced cell viability [33]. However, other studies have reported no cytotoxic effects of AgNPs on HUVEC viability [18,34]. These discrepancies may stem from differences in AgNP administration methods and the absence of ECM in the referenced studies.

A key factor underlying AgNP cytotoxicity is the induction of oxidative stress. Endothelial cells primarily produce nitric oxide (NO) via nitric oxide synthase activity [35]. Although NO has limited reactivity, its reaction with the superoxide anion produces a reactive nitrating agent [36]. Therefore, observed differences may be due to varying levels of both NO and superoxide. Our study demonstrated that nanoplatforms incorporating an ECM and AgNPs at 0.5 and 10 mg/L increased NO levels while decreasing superoxide levels. According to the literature, significant cumulative ROS production can occur after exposure to low doses of AgNPs [37]. The observed changes in mitochondrial superoxide and NO levels suggest that AgNPs induce oxidative stress in HUVECs, increasing mitochondrial superoxide production. In response, cells activate protective mechanisms, including NO production, which reduces superoxide levels [38]. Our results indicate that HUVECs respond to AgNP-induced oxidative stress by activating defense pathways to mitigate toxicity. Similar responses have been observed in other cell types, where AgNPs generate ROS, such as superoxide anions (O_2_^−^), leading to cellular damage [39].

Endothelial cell migration involves three primary mechanisms: chemotaxis (migration toward soluble chemoattractant gradients), haptotaxis (migration toward immobilized ligand gradients), and mechanotaxis (migration in response to mechanical forces) [40,41]. Although various biological factors influence angiogenesis, VEGF is considered the most critical [42]. Machado et al. [43] virtually analyzed endothelial cell migration driven by VEGF-induced chemotaxis and ECM-induced haptotaxis. The VEGF diffusion gradient is crucial for capillary formation and angiogenesis [44,45].

Our findings regarding migration were somewhat unexpected. The literature suggests that AgNPs inhibit VEGF-induced proliferation, migration, and tube formation [44,45,46], though these effects are typically observed at higher concentrations. In contrast, our results show that ECM-embedded AgNPs at 0.5 mg/L increased HUVEC migration, while at 10 mg/L, migration remained unchanged (Figure 5). Recent studies have also reported the enhanced proliferation and migration of HUVECs and human keratinocytes following AgNP exposure [47,48]. Additionally, AgNPs have been shown to upregulate VEGF expression both in vitro and in vivo, particularly in green-synthesized forms [46,48,49]. The protein corona formed during green synthesis may mimic the nanoplatform used in our study, limiting silver ion release. Furthermore, immobilized AgNPs on the ECM may act as ligands that promote haptotaxis, thus stimulating migration. This enhanced migration could explain the increased surface coverage (Figure 4) and more uniform monolayer formation. AgNPs may also promote cell adhesion [50], contributing further to the observed increase in surface coverage. Moreover, in our study, nitric oxide (NO) levels were upregulated, while superoxide levels were significantly downregulated in HUVECs exposed to the ECM containing 0.5 mg/L AgNPs. This redox shift is crucial, as NO is a well-established mediator of endothelial cell migration [51], survival [52], and barrier function [53], independent of classical growth factor pathways, such as VEGF or bFGF. The decrease in superoxide also suggests reduced oxidative stress, which may further support cytoskeletal stabilization and enhanced motility.

To address the observed pro-migratory effects at 0.5 mg/L AgNPs, it is important to consider the role of the ERK/mitogen-activated protein kinase (MAPK) signaling pathway. Cell migration and adhesion are closely associated with the activation of this pathway [54]. Within the MAPK family, the JNK/stress-activated protein kinase and p38 MAPK pathways may also mediate cellular responses to AgNP-induced stress. Notably, a proteomic analysis revealed the downregulation of growth factors, such as EGF, FGF, and PDGF, which are key upstream activators of the ERK1/2 cascade [55]. While p38 MAPK is generally activated by various stress stimuli, its activation is known to be cell-type dependent [56]. Once activated, p38 can phosphorylate downstream targets, such as MK2, MK3, and MK5 [57], potentially leading to the inactivation of GSK3β through phosphorylation at Ser389. This, in turn, promotes β-catenin accumulation and nuclear translocation, triggering gene expression through TCF/LEF transcription factors as a part of the Wnt/β-catenin signaling pathway [58]. At elevated AgNP concentrations (10 mg/L, Figure 2A,B), a marked decrease in cell viability and proliferation was observed. This shift in response may involve the activation of the JNK signaling cascade, which is recognized as a part of the stress-activated protein kinase (SAPK) family. JNK pathways respond to both internal and external stressors—such as oxidative stress induced by high levels of AgNPs—and are implicated in the regulation of proliferation, metabolism, DNA repair, and apoptosis [59].

The migration assay, which involved seeding cells at low densities on an AgNP-enriched ECM layer, enabled the simultaneous evaluation of both migration and anchorage-dependent differentiation into tubular structures—an important indicator of endothelial angiogenic potential [60]. A greater number of cellular connections (Figure 5D) and their prolonged stability (Figure 5A) suggest that the ECM enriched with low concentrations of AgNPs may enhance vessel stability. These findings emphasize the critical role of the nanoparticle concentration and the exposure mode when designing nanoparticle-modified hydrogels or scaffolds. While even low concentrations of directly applied AgNPs (e.g., in hydrocolloids) are known to be cytotoxic [61], the immobilization of AgNPs within a matrix, as demonstrated in this study, stimulated cell migration and stable monolayer formation. Similar effects on vessel maturation and regenerative processes were observed by Li et al., who used chitosan-based hydrogels [62]. In addition to their antibacterial properties, these features make AgNP-embedded hydrogels promising candidates for treating chronic and extensive wounds [63].

Changes in cellular behavior are often associated with alterations in the expression of specific proteins. Therefore, the expression levels of 43 proteins were assessed. The analysis revealed that HUVECs cultured on nanoplatforms composed of an ECM and AgNPs exhibited the lower expression of proteins involved in regulating endothelial cell behavior. This contrasts with findings by Kang et al., who reported VEGF upregulation under similar conditions [63]. However, our results are consistent with other published studies [44,64].

The analysis further showed that AgNPs can reduce the basic fibroblast growth factor (bFGF) expression, a key regulator of various biological functions, including cell survival, differentiation, proliferation, and migration [65]. This finding aligns with our proliferation assay results, which showed decreased proliferation with increasing concentrations of AgNPs. Additionally, a reduction in the expression of PECAM-1 was observed. According to the literature, PECAM-1 is highly expressed at endothelial cell–cell junctions, where it plays a critical role in restoring vascular permeability following stress [66]. Under physiological conditions, urokinase-type plasminogen activator receptor (uPAR) is typically expressed at low levels, which is consistent with our observations. However, its expression may transiently increase during inflammation, tissue remodeling, and wound healing—conditions that can lead to ECM degradation and enhanced cell migration [67]. Moreover, it is plausible that the observed downregulation of angiogenesis-related proteins reflects a negative feedback mechanism or a shift toward a more stabilized, less proliferative endothelial phenotype—one that favors organized migration and monolayer restoration over sprouting angiogenesis.

A stable, uniform monolayer formation may also be associated with an elevated level of nitric oxide (NO) (Figure 3A). Synthesized from L-arginine, NO activates soluble guanylyl cyclase, which in turn generates cyclic guanosine monophosphate (cGMP), causing vasodilation [68]. This mechanism may be linked to the reduced PECAM-1 levels observed. The increase in cGMP production can modulate cell functions via the activation of intracellular receptor proteins, including cGMP-regulated cyclic nucleotide phosphodiesterases (PDEs), cGMP-regulated ion channels, and cGMP-dependent protein kinases (PKGs). The activation of the cGMP pathway participates in a broad signaling network that induces various biological effects, both through phosphorylation-dependent and phosphorylation-independent mechanisms [69].

## 4. Materials and Methods

### 4.1. Physicochemical Analysis of AgNPs

Silver nanoparticles (AgNPs) were obtained from Nano-Tech (aXonnite, Warsaw, Poland) as a hydrocolloid with a concentration of 100 mg/L. The nanoparticles were produced by a non-explosive, high-voltage method, using a high-purity metal (99.9%) and demineralized water. The nanoparticles were sonicated and diluted in ultrapure water to working concentrations of 0.1, 0.5, 1, 5, and 10 mg/L. The zeta potential was measured by laser Doppler electrophoresis using the Smoluchowski approximation with a Zetasizer Nano-ZS90 analyzer (Malvern, Worcestershire, UK). Samples were sonicated in a cup horn of a VC 505 sonicator (Sonics & Materials, Newtown, CT, USA) for 60 s at 32 kHz amplitude, stabilized for 120 s at room temperature (25 °C), and measured in quadruplicate. The results are presented as mean ± standard deviation. The UV–Vis spectrum was measured using a Thermo Scientific NanoDrop One Spectrophotometer (Thermo Fisher Scientific, Waltham, MA, USA). Measurements were conducted across a wavelength range of 190 to 850 nm, with a step size of 0.5 nm.

AgNP morphology was visualized using a JEM-1220 transmission electron microscope (TEM; JEOL, Tokyo, Japan) operating at 80 keV and equipped with a Morada 11-megapixel camera (Olympus Soft Imaging Solutions, Münster, Germany).

### 4.2. Cell Lines

Human umbilical vein endothelial cells (HUVECs) were obtained from Thermo Fisher Scientific. The cells were maintained in Human Large Vessel Endothelial Cell Basal Medium (formerly Medium 200, Phenol Red-Free), supplemented with Large Vessel Endothelial Supplement and 1% penicillin/streptomycin (Thermo Fisher Scientific). The cultures were kept at 37 °C in a humidified atmosphere containing 5% CO_2_. The cells were used for experiments up to the sixth passage.

### 4.3. Analysis of Nanoparticles Toxicity

HUVEC viability and proliferation on ECM-coated surfaces, with or without AgNPs, was assessed using the PrestoBlue and CyQUANT assays (Thermo Fisher Scientific). The Geltrex LDEV-Free Reduced Growth Factor Basement Membrane Matrix (Thermo Fisher Scientific) was used for tissue plate coating.

The cells were seeded into 96-well plates at 5 × 10^3^ cells/well for the PrestoBlue assay and 1 × 10^4^ cells/well for the CyQUANT assay, using an ECM either without AgNPs (control) or enriched with AgNPs at final concentrations of 0.1, 0.5, 1, 5, or 10 mg/L. Blank samples were prepared for each group. After 24 h of incubation, cell viability was assessed using the PrestoBlue reagent, incubated with cells for 2 h. Then, 50 µL of medium was transferred to a separate 96-well plate for fluorescence measurement (excitation λ = 560 nm, emission λ = 590 nm) using a Tecan Infinite 200 microplate reader (Tecan Group Ltd., Männedorf, Switzerland). The viability was expressed relative to fluorescence values after subtracting the background from blanks. The assay was repeated twice, with at least five replicates per group.

For the CyQUANT proliferation assay, the cells were incubated for 48 h, after which the medium was removed by inversion and blotting onto paper towels. The plates were frozen at −70 °C, thawed the next day at room temperature, and treated with 200 µL of reaction mixture for DNA labeling. After 5 min, fluorescence was measured (excitation λ = 480 nm, emission λ = 520 nm) using a Tecan Infinite 200 microplate reader. Proliferation was expressed as the relative fluorescence after background subtraction. The experiment was performed three times, with a minimum of five replicates per condition.

### 4.4. Nitric Oxide and Mitochondrial Superoxide Production

To evaluate nitric oxide (NO) synthesis and superoxide generation, the Nitric Oxide Synthase Detection System (Sigma-Aldrich, St. Louis, MO, USA) and MitoSOX Red (Invitrogen, Thermo Fisher Scientific) were used. HUVECs were seeded in 96-well plates at a density of 1 × 10^4^ cells/well on ECM platforms containing 0.5 or 10 mg/l AgNPs. Analyses were conducted after 24 h. NO and superoxide detection were measured sequentially, with separate samples prepared for each analysis.

For NO detection, the culture medium was replaced with 200 µL of reaction mixture (prepared per manufacturer’s protocol). The cells were incubated for 2 h at room temperature in the dark, and the fluorescence was measured (excitation λ = 485 nm, emission λ = 530 nm) using a microplate reader. NO levels were expressed as relative fluorescence after blank subtraction.

For mitochondrial superoxide detection, 100 µL of 0.5 µM MitoSOX Red in Hank’s Balanced Salt Solution (HBSS, Thermo Fisher Scientific) was added to each well, followed by 30 min of incubation at 37 °C in 5% CO_2_. The cells were washed three times with HBSS before fluorescence measurement (excitation λ = 510 nm, emission λ = 580 nm). Superoxide levels were expressed as the relative fluorescence after background subtraction. Each analysis was performed in two independent experiments, with eight replicates per group.

### 4.5. HUVEC Confluence Level Analysis

To assess confluence, HUVECs (1 × 10^4^ cells) were seeded on glass-bottom dishes (18 mm growth area) coated with Geltrex either without (control) or with 0.5 mg/L AgNPs. The cells were cultured for 96 h, with the medium being refreshed after 48 h. After incubation, the cells were fixed with 4% paraformaldehyde (Sigma-Aldrich). The nuclei were stained with Hoechst 33,342 (1 μg/mL, 10 min, room temperature), and the actin cytoskeleton was labeled with Atto 633-conjugated phalloidin (Sigma-Aldrich). Images were acquired using a FV1000 confocal microscope (Olympus Corporation, Tokyo, Japan) with a 10× objective. The cell growth area and nuclear count were quantified using ImageJ software version 1.54p [70]. For nuclei quantification, images were thresholded, separated using the “watershed” function, and analyzed with the “analyze particles” tool. The data are presented as the average growth area and the nuclei count per cm^2^ from six fields of view per group.

### 4.6. Migration Analysis

Cell migration was evaluated using time-lapse cell tracking. HUVECs were stained with Hoechst 33,342 (0.5 μg/mL, 10 min, room temperature) and seeded at 1 × 10^4^ cells per dish in a growth medium on ECM-coated, glass-bottom dishes (Eppendorf, Hamburg, Germany) containing AgNPs at 0.5 and 10 mg/L or without nanoparticles (control).

The cells were imaged every 7 min over 15 h using a FV1000 confocal microscope equipped with a motorized stage and a temperature- and atmosphere-controlled chamber (37 °C, 5% CO_2_). The images were acquired using Nomarski interference contrast, and the nuclear fluorescence was captured at excitation λ = 361 nm and emission λ = 486 nm.

Migration and cell merging were assessed by tracking nuclei displacement using the TrackMate plugin in ImageJ [71]. To eliminate image drift unrelated to cell migration, image sequences were stabilized using the StackReg plugin [72]. The data are presented as the average total distance traveled per cell and the number of merge events per track, averaged across nine fields of view for each group.

### 4.7. Antibody Array Analysis

The human angiogenesis antibody array (ab193655, Abcam, Cambridge, UK) was used to evaluate the expression levels of 43 angiogenesis-related proteins. A complete array map is provided in the Supplementary Materials (Tables S1 and S2). HUVEC lysates were prepared from cells seeded in one 6-well plate per group on an ECM without AgNPs (control) or containing 0.5 mg/L AgNPs, after 24 h of incubation. The cells were washed with PBS and lysed directly in an ice-cold RIPA buffer (Thermo Fisher Scientific) supplemented with Halt Protease & Phosphatase Inhibitor Cocktail (Thermo Fisher Scientific) and 5 mM EDTA. All the subsequent steps were carried out on ice. Lysates were sonicated for 1 min (5 s intervals at 20 Hz), incubated for 30 min with vortexing every 10 min, and centrifuged at 12,000× *g* for 30 min. The supernatants were collected in fresh tubes.

The protein concentration was determined using the Pierce BCA Protein Assay Kit (Thermo Fisher Scientific) and normalized across the samples. Each membrane was incubated overnight with 200 µg of total protein. Signal detection was performed using chemiluminescence on the Azure Biosystems C400 imaging system (Azure, Dublin, CA, USA). Signal intensities were quantified and normalized to the control group using ImageJ software [71].

### 4.8. Statistical Analysis

Data from cell viability, proliferation, nitric oxide, and mitochondrial superoxide production were analyzed using a one-way analysis of variance (ANOVA) in GraphPad Prism 8 (San Diego, CA, USA). Differences between the control and treatment groups were assessed using Dunnett’s post hoc test. Other analyses (confluence, migration, and protein expression) were evaluated using one-way ANOVAs and Tukey’s HSD post hoc test for multiple comparisons. The results are presented as the mean values ± standard deviation (SD). Statistical significance was considered at *p* < 0.05.

## 5. Conclusions

AgNPs embedded in the ECM at low concentrations (0.1 to 1 mg/L) exhibited no or limited cytotoxicity, as indicated by the metabolic activity of the HUVECs. Cell proliferation decreased in a dose-dependent manner with the increasing AgNP concentration. After 24 h of incubation on the nanoplatforms, a dose-dependent increase in nitric oxide (NO) levels was observed, while mitochondrial superoxide levels decreased regardless of the AgNP concentration. These findings suggest that endothelial cells may activate defense mechanisms, including NO production, which can reduce superoxide levels.

Furthermore, the analyses revealed that AgNPs at a concentration of 0.5 mg/L enhanced cell coverage, migration, and promoted monolayer formation. This effect may be linked to elevated NO levels, which activate guanylyl cyclase, leading to vasodilation. This process may also be associated with the observed PECAM-1 expression reduction following exposure to AgNP–ECM nanoplatforms.

## Data Availability

The datasets analyzed during the current study are available from the corresponding author on reasonable request.

## Supplementary Materials

The following supporting information can be downloaded at https://www.mdpi.com/article/10.3390/ijms26104761/s1.

## Abbreviations

The following abbreviations are used in this manuscript:

AgNPs	silver nanoparticles
bFGF	basic fibroblast growth factor
cGMP	cyclic guanosine monophosphate
ECM	extracellular matrix
EDTA	ethylenediaminetetraacetic acid tetrasodium salt dihydrate
FGF	fibroblast growth factor
HBSS	Hank’s Balanced Salt Solution
HUVEC	human umbilical vein endothelial cells
MAPK	mitogen-activated protein kinase
NOs	nitric oxide
uPAR	urokinase plasminogen activator surface receptor
PBS	phosphate-buffered saline
PDEs	cGMP-regulated cyclic nucleotide phosphodiesterases
PECAM-1	platelet and endothelial cell adhesion molecule 1
PKGs	cgmp-dependent protein kinases
RANTES	regulated on activation, normal T-cell expressed and secreted chemokine
RIPA	radio-immunoprecipitation assay
ROS	reactive oxygen species
SAPKs	stress-activated protein kinases
TEM	transmission electron microscopy
TGF-β	transforming growth factor beta
TIE-2	angiopoetin-1 receptor
VEGF	vascular endothelial growth factor
VEGF-D	vascular endothelial growth factor D

## References

[1] Teleanu R.I., Chircov C., Grumezescu A.M. (2019). Tumor Angiogenesis and Anti-Angiogenic Strategies for Cancer Treatment. J. Clin. Med..

[2] Li J., Zhang Y.P., Kirsner R.S. (2003). Angiogenesis in Wound Repair: Angiogenic Growth Factors and the Extracellular Matrix. Microsc. Res. Tech..

[3] Senger D.R., Davis G.E. (2011). Angiogenesis. Cold Spring Harb. Perspect. Biol..

[4] Aplin A.E., Short S.M., Juliano R.L. (1999). Anchorage-Dependent Regulation of the Mitogen-Activated Protein Kinase Cascade by Growth Factors Is Supported by a Variety of Integrin α Chains. J. Biol. Chem..

[5] Assoian R.K., Schwartz M.A. (2001). Coordinate Signaling by Integrins and Receptor Tyrosine Kinases in the Regulation of G1 Phase Cell-Cycle Progression. Curr. Opin. Genet. Dev..

[6] Perruzzi C.A., De Fougerolles A.R., Koteliansky V.E., Whelan M.C., Westlin W.F., Senger D.R. (2003). Functional Overlap and Cooperativity among Av and B1 Integrin Subfamilies during Skin Angiogenesis. J. Investig. Dermatol..

[7] Ausprunk D.H., Folkman J. (1977). Migration and Proliferation of Endothelial Cells in Preformed and Newly Formed Blood Vessels during Tumor Angiogenesis. Microvasc. Res..

[8] Senger D.R., Perruzzi C.A., Streit M., Koteliansky V.E., De Fougerolles A.R., Detmar M. (2002). The A1β1 and A2β1 Integrins Provide Critical Support for Vascular Endothelial Growth Factor Signaling, Endothelial Cell Migration, and Tumor Angiogenesis. Am. J. Pathol..

[9] Azizi-Lalabadi M., Garavand F., Jafari S.M. (2021). Incorporation of silver nanoparticles into active antimicrobial nanocomposites: Release behavior, analyzing techniques, applications, and safety issues. Adv. Colloid Interface Sci..

[10] Munir M.U., Ahmed A., Usman M., Salman S. (2020). Recent advances in nanotechnology-aided materials in combating microbial resistance and functioning as antibiotics substitutes. Int. J. Nanomed..

[11] Kowalczyk P., Szymczak M., Maciejewska M., Laskowski Ł., Laskowska M., Ostaszewski R., Skiba G., Franiak-Pietryga I. (2021). All that glitters is not silver—A new look at microbiological and medical applications of silver nanoparticles. Int. J. Mol. Sci..

[12] Li S., Liu Y., Huang Z., Kou Y., Hu A. (2020). Efficacy and safety of nano-silver dressings combined with recombinant human epidermal growth factor for deep second-degree burns: A meta-analysis. Burns.

[13] Farouk M.M., El-Molla A., Salib F.A., Soliman Y.A., Shaalan M. (2020). The role of silver nanoparticles in a treatment approach for multidrug-resistant salmonella species isolates. Int. J. Nanomed..

[14] Ahamed M., AlSalhi M., Siddiqui M. (2010). Silver nanoparticle applications and human health. Clin. Chim. Acta.

[15] Panja A., Mishra A.K., Dash M., Pandey N.K., Singh S.K., Kumar B. (2021). Silver nanoparticles—A review. Eurasian J. Med. Oncol..

[16] Morozova O.V. (2021). Silver nanostructures: Limited sensitivity of detection, toxicity and anti-inflammation effects. Int. J. Mol. Sci..

[17] Hung H.S., Chang K.B., Tang C.M., Ku T.R., Kung M.L., Yu A.Y., Shen C.C., Yang Y.C., Hsieh H.H., Hsu S.H. (2021). Anti-Inflammatory Fibronectin-AgNP for Regulation of Biological Performance and Endothelial Differentiation Ability of Mesenchymal Stem Cells. Int. J. Mol. Sci..

[18] Sun X., Shi J., Zou X., Wang C., Yang Y., Zhang H. (2016). Silver Nanoparticles Interact with the Cell Membrane and Increase Endothelial Permeability by Promoting VE-Cadherin Internalization. J. Hazard. Mater..

[19] Murugan K., Choonara Y.E., Kumar P., Bijukumar D., du Toit L.C., Pillay V. (2015). Parameters and Characteristics Governing Cellular Internalization and Trans-Barrier Trafficking of Nanostructures. Int. J. Nanomed..

[20] Akter M., Sikder M.T., Rahman M.M., Ullah A.K.M.A., Hossain K.F.B., Banik S., Hosokawa T., Saito T., Kurasaki M. (2018). A Systematic Review on Silver Nanoparticles-Induced Cytotoxicity: Physicochemical Properties and Perspectives. J. Adv. Res..

[21] Wu M., Guo H., Liu L., Liu Y., Xie L. (2019). Size-Dependent Cellular Uptake and Localization Profiles of Silver Nanoparticles. Int. J. Nanomed..

[22] Gliga A.R., Skoglund S., Odnevall Wallinder I., Fadeel B., Karlsson H.L. (2014). Size-Dependent Cytotoxicity of Silver Nanoparticles in Human Lung Cells: The Role of Cellular Uptake, Agglomeration and Ag Release. Part. Fibre Toxicol..

[23] Behr J.-P. (1997). The Proton Sponge: A Trick to Enter Cells the Viruses Did Not Exploit. Chimia.

[24] Fahrenkrog B., Aebi U. (2003). The Nuclear Pore Complex: Nucleocytoplasmic Transport and Beyond. Nat. Rev. Mol. Cell Biol..

[25] Kim H.R., Kim M.J., Lee S.Y., Oh S.M., Chung K.H. (2011). Genotoxic Effects of Silver Nanoparticles Stimulated by Oxidative Stress in Human Normal Bronchial Epithelial (BEAS-2B) Cells. Mutat. Res.-Genet. Toxicol. Environ. Mutagen..

[26] Hackenberg S., Scherzed A., Kessler M., Hummel S., Technau A., Froelich K., Ginzkey C., Koehler C., Hagen R., Kleinsasser N. (2011). Silver Nanoparticles: Evaluation of DNA Damage, Toxicity and Functional Impairment in Human Mesenchymal Stem Cells. Toxicol. Lett..

[27] AshaRani P.V., Hande M.P., Valiyaveettil S. (2009). Anti-Proliferative Activity of Silver Nanoparticles. BMC Cell Biol..

[28] Asharani P.V., Low G., Mun K., Hande M.P., Valiyaveettil S. (2009). Cytotoxicity and Genotoxicity of Silver. ACS Nano.

[29] Yang E.J., Kim S., Kim J.S., Choi I.H. (2012). Inflammasome Formation and IL-1β Release by Human Blood Monocytes in Response to Silver Nanoparticles. Biomaterials.

[30] Jiang X., Foldbjerg R., Miclaus T., Wang L., Singh R., Hayashi Y., Sutherland D., Chen C., Autrup H., Beer C. (2013). Multi-Platform Genotoxicity Analysis of Silver Nanoparticles in the Model Cell Line CHO-K1. Toxicol. Lett..

[31] Reidy B., Haase A., Luch A., Dawson K.A., Lynch I. (2013). Mechanisms of Silver Nanoparticle Release, Transformation and Toxicity: A Critical Review of Current Knowledge and Recommendations for Future Studies and Applications. Materials.

[32] Ahamed M., Karns M., Goodson M., Rowe J., Hussain S.M., Schlager J.J., Hong Y. (2008). DNA Damage Response to Different Surface Chemistry of Silver Nanoparticles in Mammalian Cells. Toxicol. Appl. Pharmacol..

[33] Zhang T., Wang L., Chen Q., Chen C. (2014). Cytotoxic Potential of Silver Nanoparticles. Yonsei Med. J..

[34] Alkan H., Ciğerci İ.H., Ali M.M., Hazman O., Liman R., Colă F., Bonciu E. (2022). Cytotoxic and Genotoxic Evaluation of Biosynthesized Silver Nanoparticles Using Moringa Oleifera on MCF-7 and HUVEC Cell Lines. Plants.

[35] Sohn H.Y., Krotz F., Zahler S., Gloe T., Keller M., Theisen K., Schiele T.M., Klauss V., Pohl U. (2003). Crucial Role of Local Peroxynitrite Formation in Neutrophil-Induced Endothelial Cell Activation. Cardiovasc. Res..

[36] Zuberek M., Paciorek P., Bartosz G., Grzelak A. (2017). Silver Nanoparticles Can Attenuate Nitrative Stress. Redox Biol..

[37] Shi J., Sun X., Lin Y., Zou X., Li Z., Liao Y., Du M., Zhang H. (2014). Endothelial Cell Injury and Dysfunction Induced by Silver Nanoparticles through Oxidative Stress via IKK/NF-ΚB Pathways. Biomaterials.

[38] Redolfi-Bristol D., Yamamoto K., Marin E., Zhu W., Mazda O., Riello P., Pezzotti G. (2024). Exploring the Cellular Antioxidant Mechanism against Cytotoxic Silver Nanoparticles: A Raman Spectroscopic Analysis. Nanoscale.

[39] Rai M., Yadav A., Gade A. (2009). Silver Nanoparticles as a New Generation of Antimicrobials. Biotechnol. Adv..

[40] Gov N.S. (2009). Traction forces during collective cell motion. HFSP J..

[41] Lamalice L., Le Boeuf F., Huot J. (2007). Endothelial cell migration during angiogenesis. Circ Res..

[42] Guerra A., Belinha J., Natal Jorge R. (2020). A preliminary study of endothelial cell migration during angiogenesis using a meshless method approach. Int. J. Numer. Method Biomed. Eng..

[43] Machado M.J., Watson M.G., Devlin A.H., Chaplain M.A., McDougall S.R., Mitchell C.A. (2011). Dynamics of Angiogenesis During Wound Healing: A Coupled In Vivo and In Silico Study. Microcirculation.

[44] Gurunathan S., Lee K.J., Kalishwaralal K., Sheikpranbabu S., Vaidyanathan R., Eom S.H. (2009). Antiangiogenic properties of silver nanoparticles. Biomaterials.

[45] Kalishwaralal K., Banumathi E., Pandian S.R.K., Deepak V., Muniyandi J., Eom S.H., Gurunathan S. (2009). Silver nanoparticles inhibit VEGF induced cell proliferation and migration in bovine retinal endothelial cells. Colloids Surf. B Biointerfaces.

[46] Lu C., Liu Y., Liu Y., Kou G., Chen Y., Wu X., Lv Y., Cai J., Chen R., Luo J. (2023). Silver Nanoparticles Cause Neural and Vascular Disruption by Affecting Key Neuroactive Ligand-Receptor Interaction and VEGF Signaling Pathways. Int. J. Nanomed..

[47] Palanisamy C.P., Poompradub S., Sansanaphongpricha K., Jayaraman S., Subramani K., Sonsudin F. (2024). Increased expression levels of PDGF and VEGF magnify the wound healing potential facilitated by biogenic synthesis of silver nanoparticles. Nano-Struct. Nano-Objects.

[48] Rama P., Baldelli A., Vignesh A., Altemimi A.B., Lakshmanan G., Selvam R., Arunagirinathan N., Murugesan K., Pratap-Singh A. (2022). Antimicrobial, antioxidant, and angiogenic bioactive silver nanoparticles produced using *Murraya paniculata* (L.) jack leaves. Nanomater. Nanotechnol..

[49] Mostafavi S., Naderi Soorki M., Kesmati M., Dorostghoal M., Andashti B. (2024). Molecular effect of Silver nanoparticles on wound healing activity of Salvia officinalis extract in adult mice. Nanomed. Res. J..

[50] Arsenopoulou Z.V., Taitzoglou I.A., Molyvdas P.A., Gourgoulianis K.I., Hatzoglou C., Zarogiannis S.G. (2018). Silver Nanoparticles Alter Cell Adhesion and Proliferation of Sheep Primary Mesothelial Cells. In Vivo.

[51] Goligorsky M.S., Budzikowski A.S., Tsukahara H., Noiri E. (1999). Co-Operation between Endothelin and Nitric Oxide in Promoting Endothelial Cell Migration and Angiogenesis. Clin. Exp. Pharmacol. Physiol..

[52] Dimmeler S., Zeiher A.M. (1999). Nitric Oxide—An Endothelial Cell Survival Factor. Cell Death Differ..

[53] Boje K.M.K. (1996). Inhibition of Nitric Oxide Synthase Attenuates Blood-Brain Barrier Disruption during Experimental Meningitis. Brain Res..

[54] Agostinis C., Bulla R., Tripodo C., Gismondi A., Stabile H., Bossi F., Guarnotta C., Garlanda C., De Seta F., Spessotto P. (2010). An Alternative Role of C1q in Cell Migration and Tissue Remodeling: Contribution to Trophoblast Invasion and Placental Development. J. Immunol..

[55] Guo Y., Pan W., Liu S., Shen Z., Xu Y., Hu L. (2020). ERK/MAPK Signalling Pathway and Tumorigenesis (Review). Exp. Ther. Med..

[56] Han J., Wu J., Silke J. (2020). An Overview of Mammalian P38 Mitogen-Activated Protein Kinases, Central Regulators of Cell Stress and Receptor Signaling. F1000Research.

[57] New L., Jiang Y., Zhao M., Liu K., Zhu W., Flood L.J., Kato Y., Parry G.C.N., Han J. (1998). PRAK, a Novel Protein Kinase Regulated by the P38 MAP Kinase. EMBO J..

[58] Liu J., Xiao Q., Xiao J., Niu C., Li Y., Zhang X., Zhou Z., Shu G., Yin G. (2022). Wnt/β-Catenin Signalling: Function, Biological Mechanisms, and Therapeutic Opportunities. Signal Transduct. Target. Ther..

[59] Johnson G.L., Nakamura K. (2007). The C-Jun Kinase/Stress-Activated Pathway: Regulation, Function and Role in Human Disease. Biochim. Biophys. Acta-Mol. Cell Res..

[60] Faulkner A., Purcell R., Hibbert A., Latham S., Thomson S., Hall W.L., Wheeler-Jones C., Bishop-Bailey D. (2014). A thin layer angiogenesis assay: A modified basement matrix assay for assessment of endothelial cell differentiation. BMC Cell Biol..

[61] He J., Ma Y., Niu X., Pei J., Yan R., Xu F., Ma J., Ma X., Jia S., Ma W. (2024). Silver nanoparticles induce endothelial cytotoxicity through ROS-mediated mitochondria-lysosome damage and autophagy perturbation: The protective role of N-acetylcysteine. Toxicology.

[62] Li S., Xu Y., Zheng L., Fan C., Luo F., Zou Y., Li X., Zha Z.G., Zhang H.T., Wang X. (2025). High Self-Supporting Chitosan-Based Hydrogel Ink for In Situ 3D Printed Diabetic Wound Dressing. Adv. Funct. Mater..

[63] Kang K., Lim D.H., Choi I.H., Kang T., Lee K., Moon E.Y., Yang Y., Lee M.S., Lim J.S. (2011). Vascular Tube Formation and Angiogenesis Induced by Polyvinylpyrrolidone-Coated Silver Nanoparticles. Toxicol. Lett..

[64] Saeed B.A., Lim V., Yusof N.A., Khor K.Z., Rahman H.S., Samad N.A. (2019). Antiangiogenic Properties of Nanoparticles: A Systematic Review. Int. J. Nanomed..

[65] Yun Y.R., Won J.E., Jeon E., Lee S., Kang W., Jo H., Jang J.H., Shin U.S., Kim H.W. (2010). Fibroblast Growth Factors: Biology, Function, and Application for Tissue Regeneration. J. Tissue Eng..

[66] Lertkiatmongkol P., Liao D., Mei H., Hu Y., Newman P.J. (2016). Endothelial Functions of Platelet/Endothelial Cell Adhesion Molecule-1 (CD31). Curr. Opin. Hematol..

[67] Zhai B.T., Tian H., Sun J., Zou J.B., Zhang X.F., Cheng J.X., Shi Y.J., Fan Y., Guo D.Y. (2022). Urokinase-Type Plasminogen Activator Receptor (UPAR) as a Therapeutic Target in Cancer. J. Transl. Med..

[68] Villar I.C., Francis S., Webb A., Hobbs A.J., Ahluwalia A. (2006). Novel Aspects of Endothelium-Dependent Regulation of Vascular Tone. Kidney Int..

[69] Tousoulis D., Kampoli A.-M., Tentolouris Nikolaos Papageorgiou C., Stefanadis C. (2011). The Role of Nitric Oxide on Endothelial Function. Curr. Vasc. Pharmacol..

[70] Schneider C.A., Rasband W.S., Eliceiri K.W. (2012). NIH Image to ImageJ: 25 years of image analysis. Nat. Methods.

[71] Tinevez J.Y., Perry N., Schindelin J., Hoopes G.M., Reynolds G.D., Laplantine E., Bednarek S.Y., Shorte S.L., Eliceiri K.W. (2017). TrackMate: An open and extensible platform for single-particle tracking. Methods.

[72] Thevenaz P., Ruttimann U.E., Unser M. (1998). A pyramid approach to subpixel registration based on intensity. IEEE Trans. Image Process..

