# Supplementary material for: Silver Nanoparticles at Low Concentrations Embedded in ECM Promote Endothelial Monolayer Formation and Cell Migration
Source: Int J Mol Sci. 2025 May 16;26(10):4761. doi: 10.3390/ijms26104761 (PMC12112687; doi:10.3390/ijms26104761)
Supplement: Supplementary file 1 [file ijms-26-04761-s001.zip › ijms-3619834-supplementary.pdf]

Supplementary Data

Silver Nanoparticles at Low Concentrations Embedded in ECM Promote Endothelial Monolayer Formation and Cell Migration

Promote Endothelial Monolayer Formation and Cell Migration

Barbara Wójcik *, Katarzyna Zawadzka, Anna Hotowy, Maria Jóźwiak, Klaudia Jusińska and Mateusz Wierzbicki

Department of Nanobiotechnology, Warsaw University of Life Science, Ciszewskiego 8, 02-786 Warsaw, Poland; katarzyna_zawadzka1@sggw.edu.pl (K.Z.); anna_hotowy@sggw.edu.pl (A.H.); maria.jozwiak199@gmail.com (M.J.); s200008@sggw.edu.pl (K.J.); mateusz_wierzbicki@sggw.edu.pl (M.W.)

* Correspondence: barbara_wojcik1@sggw.edu.pl

Table S1. The location of particular proteins on the first membrane (M1)

**M1**	A	B	C	D	E	F	G	H
1	Pos	Pos	Neg	Neg	ANG	EGF	ENA-78	bFGF
2	Pos	Pos	Neg	Neg	ANG	EGF	ENA-78	bFGF
3	GRO	INF-Ɣ	IGF-1	IL-6	IL-8	Leptin	MCP-1	PDGF-BB
4	GRO	INF-Ɣ	IGF-1	IL-6	IL8	Leptin	MCP-1	PDGF-BB
5	PLGF	RANTES	TGF-β1	TIMP-1	TIMP-2	THPO	VEGF	VEGF-D
6	PLGF	RANTES	TGF-β1	TIMP-1	TIMP-2	THPO	VEGF	VEGF-D
7	BLANK	BLANK	BLANK	BLANK	BLANK	BLANK	Neg	Pos
8	BLANK	BLANK	BLANK	BLANK	BLANK	BLANK	Neg	Pos

Table S2. The location of particular proteins on the second membrane (M2)

**M2**	A	B	C	D	E	F	G	H
1	Pos	Pos	Neg	Neg	ANGPT1	ANGPT2	PLG	Endostatin
2	Pos	Pos	Neg	Neg	ANGPT1	ANGPT2	PLG	Endostatin
3	G-CSF	GM-CSF	I-309	IL-10	IL-1α	IL-1β	IL-2	IL-4
4	G-CSF	GM-CSF	I-309	IL-10	IL-1α	IL-1β	IL-2	IL-4
5	I-TAC	MCP-3	MCP-4	MMP-1	MMP-9	PECAM-1	TIE-2	TNF-α
6	I-TAC	MCP-3	MCP-4	MMP-1	MMP-9	PECAM-1	TIE-2	TNF-α
7	uPAR	VEGFR2	VEGFR3	BLANK	BLANK	BLANK	Neg	Pos
8	uPAR	VEGFR2	VEGFR3	BLANK	BLANK	BLANK	Neg	Pos
